# Linking of Primary Care Records to Census Data to Study the Association between Socioeconomic Status and Cancer Incidence in Southern Europe: A Nation-Wide Ecological Study

**DOI:** 10.1371/journal.pone.0109706

**Published:** 2014-10-20

**Authors:** Maria Garcia-Gil, Josep-Maria Elorza, Marta Banque, Marc Comas-Cufí, Jordi Blanch, Rafel Ramos, Leonardo Méndez-Boo, Eduardo Hermosilla, Bonaventura Bolibar, Daniel Prieto-Alhambra

**Affiliations:** 1 Research Unit, Family Medicine, Girona, Spain, and Jordi Gol Institute for Primary Care Research (IDIAP Jordi Gol), Catalunya, Spain; 2 Translab Research Group, Department of Medical Sciences, School of Medicine, University of Girona, Catalunya, Spain; 3 Jordi Gol Institute for Primary Care Research (IDIAP Jordi Gol), Catalunya, Spain; 4 Cancer Prevention Unit and Cancer Registry, Department of Epidemiology and Evaluation, Hospital del Mar, Barcelona, Catalunya, Spain; 5 Primary Care Services, Girona, Spain, and Catalan Institute of Health (ICS), Catalunya, Spain; 6 Primary Care Information System, Catalan Institute of Health (ICS), Catalunya, Spain; 7 Nuffield Department of Orthopaedics, Rheumatology and Musculoskeletal Sciences, University of Oxford, Oxford, United Kingdom; Federico II University of Naples, Italy

## Abstract

**Background:**

Area-based measures of economic deprivation are seldom applied to large medical records databases to establish population-scale associations between deprivation and disease.

**Objective:**

To study the association between deprivation and incidence of common cancer types in a Southern European region.

**Methods:**

Retrospective ecological study using the SIDIAP (Information System for the Development of Research in Primary Care) database of longitudinal electronic medical records for a representative population of Catalonia (Spain) and the MEDEA index based on urban socioeconomic indicators in the Spanish census. Study outcomes were incident cervical, breast, colorectal, prostate, and lung cancer in 2009–2012. The completeness of SIDIAP cancer recording was evaluated through linkage of a geographic data subset to a hospital cancer registry. Associations between MEDEA quintiles and cancer incidence was evaluated using zero-inflated Poisson regression adjusted for sex, age, smoking, alcoholism, obesity, hypertension, and diabetes.

**Results:**

SIDIAP sensitivity was 63% to 92% for the five cancers studied. There was direct association between deprivation and lung, colorectal, and cervical cancer: incidence rate ratios (IRR) 1.82 [1.64–2.01], IRR 1.60 [1.34–1.90], IRR 1.22 [1.07–1.38], respectively, comparing the most deprived to most affluent areas. In wealthy areas, prostate and breast cancers were more common: IRR 0.92 [0.80–1.00], IRR 0.91 [0.78–1.06]. Adjustment for confounders attenuated the association with lung cancer risk (fully adjusted IRR 1.16 [1.08–1.25]), reversed the direction of the association with colorectal cancer (IRR 0.90 [0.84–0.95]), and did not modify the associations with cervical (IRR 1.27 [1.11–1.45]), prostate (0.74 [0.69–0.80]), and breast (0.76 [0.71–0.81]) cancer.

**Conclusions:**

Deprivation is associated differently with the occurrence of various cancer types. These results provide evidence that MEDEA is a useful, area-based deprivation index for analyses of the SIDIAP database. This information will be useful to improve screening programs, cancer prevention and management strategies, to reach patients more effectively, particularly in deprived urban areas.

## Introduction

Links between morbidity, all-cause mortality, cause-specific mortality, and economic deprivation are well documented [1]–[5]; this association also applies to cancer incidence [6]–[8] and related mortality, the latter being partially explained by differences in health care provision according to socioeconomic area [8]–[9]. Although the link between deprivation and health has been associated with health-related behaviors [10], area-based socioeconomic measurements have proved useful to monitor health inequalities [11] in both geographic-area [12] and individual level studies [13]–[14].

There is a scarcity of data and lack of experience in using area-based measures of deprivation together with large, routinely collected medical records databases to establish the association between deprivation and disease at a population scale. Furthermore, the population databases often lack socioeconomic data or the reliability of the available data is uncertain. Accordingly, the construction of deprivation measures that are readily available and representative of the whole population could overcome this gap in patient data [11], [14]–[15]. Information about socioeconomic status has fundamental implications for health service provision, resource allocation, and prevention efforts.

One way to measure the extent to which these measures would be useful to detect health inequalities from an analysis of electronic medical records is to link primary care records to census data for the area served. This allows us to ascertain the relationship between the existing deprivation indices and specific health conditions. The present study aimed to analyze the association between the MEDEA deprivation index, validated for Spain, and the population incidence of five cancers (lung, colorectal, breast, prostate, and cervical) in a Southern European region (Catalonia, Spain).

## Methods

### Ethics Statement

Approval for all observational research using SIDIAP data is obtained from a local ethics committee (Clinical Research Ethics Committee of the IDIAP Jordi Gol).

### Design

Retrospective ecological study.

### Data source

The Information System for the Development of Research in Primary Care (SIDIAP) is a clinical database of anonymized patient records for nearly six million people (80% of the Catalan population and 10.2% of the total population of Spain) registered in 274 primary care practices throughout Catalonia, with a total of 3,414 general practitioners (GP) and is highly representative of both urban and rural areas [16]–[17]. The information contained in SIDIAP is collected by health professionals during routine visits over time, and then it provides a good source of longitudinal population-based data.

The information recorded includes demographic and lifestyle factors relevant to primary care settings (body mass index, smoking status, alcohol use, etc); clinical diagnoses, outcomes, and events (coded according to the International Classification of Diseases, 10th revision [ICD-10]); referrals to specialists and laboratory tests; and prescribed medications actually dispensed by community pharmacies. The quality of SIDIAP data has been previously documented, and the database has been widely used to study the epidemiology of a number of health outcomes [17]–[22].

### Main exposure: socioeconomic status

The main exposure considered for this study was the ecological MEDEA index, calculated using five census-based socioeconomic indicators (percentages, by census tract): 1) unemployment rate, 2) manual workers, 3) temporary workers, 4) illiterate adults (or less than basic, mandated education), and 5) school drop-outs among the population less than 16 years old. The MEDEA index was calculated as the weighted sum of these indicators, using the weights that correspond to the calculated values in the original MEDEA Project. Further details on the construction of the MEDEA index were previously published elsewhere [23].

The SIDIAP data were linked to the 2001 census data after harmonization of the recorded patient address information with census residence data and allocation of each individual record to the correct census tract. SIDIAP records from rural areas were excluded, as MEDEA has only been validated for urban populations. Each of the census tracts included was assigned a MEDEA index score based on the census data for its population.

### Study outcome and diagnostic validation

The study outcomes were incident cases of the five most common cancers: colorectal, breast, lung, prostate, and cervix in the period 2009–2012. Only females were included in the breast and cervical cancer analyses, and only men in the analysis of prostate cancer.

Cancers were ascertained using ICD-10 codes as recorded by primary care physicians: C50-D05 for breast, C53 for cervical, C61-D07.5 for prostate, C18-19-20-D01 for colorectal, C34-D02.2 for lung cancers. Sensitivity as a measure of the completeness of the set of codes used in SIDIAP was validated by linkage to a long-standing cancer registry at Barcelona’s Hospital del Mar [24] using a trusted third party. The 212,863 people living in the Hospital del Mar area of reference were identified and linked to the Cancer Registry for this purpose.

### Confounders and potential explanatory variables

A set of covariates were defined a priori and multivariate regression models adjusted accordingly to explore either confounding or potential causal pathways. The prevalence of the following variables was characterized at baseline (1/1/2009) at the census tract level: age (5-year groups), female sex, obesity (body mass index of 30 kg/m^2^ or above), hypertension (ICD-10 codes I10–I15), and diabetes (ICD-10 codes E10–E14), and SIDIAP codes for current smoking (yes/no), and high-risk alcohol intake (yes/no). Smoking and high-risk intake are recorded in SIDIAP according to Catalan clinical practice guidelines [25], [26].

### Statistical analysis

Variables were described as weighted means; weighted one-way ANOVA contrasts were performed to contrast statistical differences between MEDEA quintiles. Cumulative incidences and 95% confidence intervals (95%CI) for all studied cancers were also calculated. MEDEA scores were categorized by quintiles. The aggregated nature of the data resulted in a higher incidence of zeros than would expected if the data were Poisson distributed. Thus, the association between MEDEA quintile (the higher, the more deprived) and the incidence of the studied cancers in 2009–2012 was evaluated using age- and sex-adjusted, zero-inflated, Poisson regression modeling (IRR and 95%CI) [27].

We further adjusted for lifestyle factors (smoking, alcoholism, obesity) and finally fitted a multivariate model, adjusted for all these factors and further adjusted for common comorbidities (hypertension, diabetes) to study potential causal pathways. All the described analyses were repeated after stratification by sex (with the exception of prostate, cervical, and breast cancer). A clustered “sandwich” estimator was calculated to estimate the variance-covariance matrix, which allowed us to account for the correlation between individuals living in the same census tract. All analyses were performed with STATA v12 [28].

## Results

### Baseline characteristics

The study population of 3,701,169 primary care patients living in 4,096 urban census tracts was analyzed for the 2009–2012 study period. Table 1 presents census-based socioeconomic characteristics of the study population that are used to calculate the MEDEA index. The proportion of manual workers, unemployment rates, temporary workers, people with limited education (or illiteracy) and of young people (<16 years of age) not in school ranged from 29.4% to 73.6%, 8.0% to 14.6%, 14.3% to 28.6%, 19.2% to 51.2%, and from 5.6% to 24.8%, respectively. Women accounted for 51% of the total population and overall mean age was 45.4 (18.3) years. Regarding sociodemographic and clinical characteristics (Table 2), the proportion of women and mean age decreased with deprivation but the most deprived areas had the highest prevalence of lifestyle risk factors (smoking, alcoholism and obesity) and comorbidities (hypertension and diabetes).

**Table 1:** Baseline socioeconomic characteristics of study population, by MEDEA quintile.

		% Manualworkers	% Unemployed	% Temporaryworkers	% Adults with limitededucation/Illiteracy	% Young people(<16 years old) not in school
Total		54.2	10.7	20.6	35.5	13.9
MEDEA quintiles	First	29.4	8.0	14.3	19.2	5.6
	Second	46.4	9.3	17.7	29.5	9.1
	Third	57.6	10.2	20.1	36.2	12.8
	Fourth	65.9	11.9	23.2	41.5	15.8
	Fifth	73.6	14.6	28.6	51.2	24.8

All p-values (weighted one-way ANOVA contrasts) <0.0001.

**Table 2:** Baseline socio demographic and clinical characteristics of study population, by socioeconomic status.

		% Women	Age in years:mean (SD)	% Currentsmoker	% High- riskalcohol intake	% Obesity	% Hyper-tension	% Diabetes
**Total**		51.1	45.4 (18.3)	17.3	1.3	11.9	16.8	6.2
**MEDEA quintile**	First	53.3	46.9 (18.7)	13.8	1.0	7.9	15.4	4.8
	Second	52.2	46.0 (18.4)	16.3	1.2	10.7	16.7	5.8
	Third	51.3	45.7 (18.3)	17.9	1.3	12.3	17.3	6.3
	Fourth	50.6	45.3 (18.6)	18.7	1.4	13.6	17.5	6.8
	Fifth	48.3	43.6 (18.1)	19.3	1.6	14.5	16.9	7.1

All p-values (weighted one-way ANOVA contrasts) <0.0001.

### Outcome and data validation

Within the period 2009–2012, overall cumulative incidences (x 10,000 persons at risk) of lung, colorectal, prostate, breast, and cervical cancers were: 21.5 [CI 95%: 21.1–22.1], 32.9 [32.3–33.5], 57.8 [56.5–59.1], 68.3 [67.1–69.5], and 14.6 [14.1–15.2], respectively. For the subset of 212,863 people living in the Hospital del Mar area of reference, 2,284 cases of cancer were identified in both datasets (509 lung, 567 colorectal, 494 prostate, 679 breast, 35 cervical); 3,322 patients had a cancer diagnosis according to primary care records but were not included in the registry (339 lung, 1,418 colorectal, 729 prostate, 746 breast, 90 cervical); and 410 participants (144 lung, 79 colorectal, 111 prostate, 59 breast, 17 cervical) were recorded in the cancer registry but not in primary care records. The sensitivity of SIDIAP compared to the cancer registry (gold standard) was 84.7% [83.3–86.1] overall, 77.9% [74.6–80.9] for lung cancer, 87.7% [85.0–90.0] for colorectal cancer, 81.6% [78.3–84.5] for prostate cancer, 92.0% [89.8–93.7] for breast cancer, and 63.7% [53.7–78.4] for cervical cancer.

### Crude associations

Comparing the most deprived to most affluent areas, crude zero-inflated Poisson regression models showed a direct association between deprivation and lung, colorectal, and cervical cancer: incidence rate ratios (IRR) 1.82 [1.64–2.01], IRR 1.60 [1.34–1.90], IRR 1.65 [1.07–2.54], respectively. In wealthy areas, prostate and breast cancers were more common: IRR 0.92 [0.80–1.00], IRR 0.91 [0.78–1.06]. These broad observations were refined by the adjusted analyses, as described below.

### Age- and sex-adjusted associations

The observed associations between deprivation and the risk of lung and cervical cancers were partially attenuated after adjustment for age and sex IRR 1.28 [1.19–1.38] and for age only 1.23 [1.08–1.39], respectively (Tables 3 and 4). In colorectal cancers, the association was no longer significant after adjusting for differences in age and sex: IRR 1.03 [0.96–1.10] for fifth compared to first MEDEA quintiles (Table 3). The effect size of the associations between deprivation and risk of breast and prostate cancers was higher after adjusting for age: IRR 0.79 [0.74–0.84] and IRR 0.77 [0.72–0.83], respectively, for fifth compared to first MEDEA quintiles (Tables 4 and 5).

**Table 3:** Association between MEDEA quintile and incidence of lung and colorectal cancer.

MEDEA quintile	Lung cancer			Colorectal cancer		
	Crude IRR	Sex- and age- adjustedIRR	Fully-adjustedIRR	Crude IRR	Sex- and age-adjustedIRR	Fully-adjustedIRR
**Total**						
First	1	1	1	1	1	1
Second	1.12 (1.04–1.21)	1.03 (0.96–1.11)	1.00 (0.93–1.07)	1.13 (1.05–1.21)	1.02 (0.97–1.09)	0.98 (0.92–1.04)
Third	1.26 (1.17–1.37)	1.06 (0.98–1.14)	1.00 (0.93–1.08)	1.27 (1.16–1.39)	1.01 (0.95–1.07)	0.93 (0.88–0.99)
Fourth	1.40 (1.28–1.53)	1.07 (1.00–1.15)	1.00 (0.93–1.08)	1.43 (1.25–1.62)	1.00 (0.94–1.07)	0.90 (0.85–0.96)
Fifth	1.82 (1.64–2.01)	1.28 (1.19–1.38)	1.16 (1.08–1.25)	1.60 (1.34–1.90)	1.03 (0.96–1.10)	0.90 (0.84–0.95)
**Men**						
First	1	1	1	1	1	1
Second	1.17 (1.06–1.29)	1.08 (0.99–1.17)	1.04 (0.96–1.13)	1.13 (1.03–1.23)	0.99 (0.92–1.07)	0.96 (0.89–1.03)
Third	1.42 (1.25–1.61)	1.16 (1.07–1.26)	1.10 (1.01–1.19)	1.40 (1.25–1.56)	1.03 (0.96–1.11)	0.98 (0.91–1.06)
Fourth	1.59 (1.35–1.88)	1.20 (1.11–1.30)	1.11 (1.02–1.20)	1.60 (1.38–1.87)	1.02 (0.94–1.09)	0.95 (0.88–1.03)
Fifth	2.10 (1.68–2.62)	1.47 (1.35–1.59)	1.31 (1.20–1.42)	1.74 (1.41–2.15)	0.96 (0.89–1.04)	0.89 (0.82–0.97)
**Women**						
First	1	1	1	1	1	1
Second	1.01 (0.85–1.21)	0.92 (0.78–1.07)	0.91 (0.78–1.07)	1.12 (1.01–1.24)	1.04 (0.96–1.14)	1.00 (0.92–1.09)
Third	1.01 (0.76–1.34)	0.76 (0.65–0.89)	0.77 (0.65–0.90)	1.10 (0.94–1.27)	0.92 (0.85–1.01)	0.87 (0.79–0.95)
Fourth	1.23 (0.72–2.08)	0.73 (0.62–0.87)	0.73 (0.62–0.86)	1.19 (0.96–1.49)	0.92 (0.84–1.00)	0.84 (0.76–0.93)
Fifth	1.70 (0.69–4.16)	0.79 (0.66–0.93)	0.78 (0.65–0.93)	1.43 (1.06–1.94)	1.01 (0.92–1.10)	0.90 (0.82–1.00)

**Table 4:** Association between MEDEA quintile and incidence of breast and cervical cancer.

MEDEA quintiles	Breast cancer			Cervical cancer		
	Crude	Age-adjusted IRR	Fully adjusted IRR	Crude IRR	Age-adjusted IRR	Fully-adjusted IRR
First	1	1	1	1	1	1
Second	0.98 (0.92–1.04)	0.95 (0.90–1.00)	0.93 (0.88–0.99)	1.00 (0.88–1.14)	1.00 (0.88–1.14)	1.01 (0.88–1.15)
Third	0.95 (0.87–1.03)	0.87 (0.83–0.92)	0.85 (0.80–0.90)	1.10 (0.98–1.25)	1.11 (0.98–1.25)	1.11 (0.98–1.26)
Fourth	0.94 (0.84–1.05)	0.83 (0.78–0.88)	0.80 (0.75–0.85)	1.27 (1.12–1.43)	1.27 (1.13–1.44)	1.29 (1.14–1.47)
Fifth	0.91 (0.78–1.06)	0.79 (0.74–0.84)	0.76 (0.71–0.81)	1.22 (1.07–1.38)	1.23 (1.08–1.39)	1.27 (1.11–1.45)

**Table 5:** Association between MEDEA quintile and prostate cancer incidence.

MEDEA quintiles	Prostate cancer		
	Crude IRR	Age-adjusted IRR	Fully-adjusted IRR
First	1	1	1
Second	1.01 (0.94–1.09)	0.95 (0.89–1.01)	0.93 (0.88–0.99)
Third	1.01 (0.92–1.10)	0.88 (0.83–0.93)	0.86 (0.80–0.91)
Fourth	1.04 (0.93–1.16)	0.87 (0.82–0.93)	0.84 (0.79–0.90)
Fifth	0.92 (0.80–1.06)	0.77 (0.72–0.83)	0.74 (0.69–0.80)

### Full adjustment

Full adjustment for lifestyle-related cancer risk factors (smoking, alcohol use, and obesity) and common comorbidities (hypertension and diabetes mellitus) further attenuated the effect size of the association between socioeconomic status and lung cancer risk (fully adjusted IRR 1.16 [1.08–1.25]), and even reversed the direction of the association between deprivation and colorectal cancer (fully adjusted IRR 0.90 [0.84–0.95]) (Table 3). By contrast, the observed associations between MEDEA deprivation scores and the remaining cancer risks were not modified after adjustment for these same factors: fully adjusted IRR 1.27 [1.11–1.45] for cervical (Table 4), 0.74 [0.69–0.80] for prostate (Table 5), and 0.76 [0.71–0.81] for breast cancer (Table 4).

### Stratification by sex

Analyses of the relationship between socioeconomic status and lung and colorectal cancer after stratification by sex are summarized in Table 3. In summary, the effect of deprivation on risk of lung cancer was stronger in men (age-adjusted IRR 1.47 [1.35–1.59] for the most deprived compared to wealthiest areas), and the direction of the association was inverted for women (age-adjusted IRR 0.79 [0.66–0.93]). On the other hand, sex stratification did not alter the results for the association between MEDEA quintiles and colorectal cancer risk.

## Discussion

The present study showed that increasing levels of deprivation were associated with an increased incidence of lung cancer in men and of cervical cancer in the most deprived compared to the wealthiest population groups. Conversely, socioeconomic deprivation was associated with decreased incidence of prostate and breast cancers in our data.

Most of the observed association between deprivation and lung cancer is explained by differences in the age-sex composition of the study population and in the prevalence of common lifestyle-related cancer risk factors and comorbidities. Interestingly, adjustment for these same variables inverted the association between socioeconomic status and colorectal cancer: the risk of this type of cancer was 10% lower in the most deprived compared to the most wealthy areas in multivariate regression models. Adjustment for all these factors did not alter the observed associations between socioeconomic deprivation and the risks of breast, prostate, or cervical cancer.

SIDIAP provides good sensitivity for the study of the epidemiology of cancer, with highly sensitive diagnostic coding ranging from 63% (cervical) to 92% (breast cancer). Our results provide evidence that the MEDEA index is a useful area-based deprivation index for use in analysis of the SIDIAP database, although it has not been constructed for use in rural areas.

Moreover, the linkage of SIDIAP and census data proved essential to ascertain the excess risk of cancer related to social deprivation in primary care settings.

The cancer types selected for analysis have the highest incidence in Spain, and their association with risk factors closely linked to socioeconomic status (life-style factors, sexual behavior, access to private healthcare or screening uptake) is well known. Our results were consistent with previously published studies [29]–[30].

Consistent with our data, lung cancer risk increases in deprived areas [6], [30]–[32] and its incidence is highest in the most deprived men even after adjustment for smoking [33]. This excess risk might be explained by other risk factors such as exposure to occupational carcinogens or dietary factors [33], [34], not accounted for in our study design. Unexpectedly, we found an inverse association between risk of lung cancer and deprivation in women; although some studies have shown that women with greater economic deprivation have a decreased risk of dying from lung cancer [35], a number of previous studies reported that lung cancer risk increases in deprived areas regardless of sex [6], [30]–[31].

On the other hand, there is no clear association between deprivation and colorectal cancer [7], [33], [36]–[37], probably due to changes in socioeconomic patterns over recent decades. Our study found an inverse association between deprivation and prostate cancer incidence, in agreement with some other studies [38]; this can likely be explained not only by inequalities in screening (periodic prostate-specific antigen measurement or physical examination by a urologist) related to socioeconomic strata [30], [38] but also by inequalities in exposure to risk factors, such as diet [39].

Regarding cancers specific to women, cervical and breast cancer have opposite associations with socioeconomic status. Cervical cancer incidence has been consistently shown to be more common in deprived populations [6], [30], probably due to inadequate or insufficient screening as well as to possible differences in age of first sexual intercourse, number of sexual partners, or fertility rates in different social strata, leading to unequal exposure to human papilloma virus [30], [38], [40]–[41]. Conversely, women living in wealthy areas are more likely to be diagnosed with breast cancer [30], [38], [42]. This association could be explained by differences in breastfeeding habits, age of first pregnancy, and use of hormone replacement therapy [42].

### Strengths and limitations

The access to internally validated, high-quality electronic medical records provided a large sample size, reflected real-life conditions, and warranted high external validity. Furthermore, SIDIAP data are highly representative of Catalonia, including both urban and rural areas [17]. Of the covariates analysed, cardiovascular risk factors in SIDIAP have been previously validated for research use [18]. In general, smoking and alcohol intake are under-recorded in SIDIAP, compared with the Catalan National Health Survey [43]. In our results, however, the gradient and the higher prevalence of these risk factors in the most deprived population are consistent with the Health Survey results and other analyses reported in the literature [43]–[44].

The Hospital del Mar Cancer Registry is not a population registry, although it is quite representative of the population covered. Therefore, we could only calculate the sensitivity of SIDIAP for the cancers studied. Nonetheless, the relationships observed between the MEDEA index and cancer incidence were robust and in accordance with the literature. The most recent MEDEA index available was constructed using 2001 census data; this was matched to 2009–2012 cancer incidence data, on the dual assumption that cancer has a long latency period and the socioeconomic character (i.e., deprivation status) of census tracts does not vary significantly within one census period [45]. As an ecological measure of deprivation, the MEDEA index is more likely to be prone to misclassification [46] than are individual measures. However, the resulting bias is towards the null hypothesis because the misclassification is probably not related to the outcome. In addition, economic heterogeneity within the MEDEA strata may result in a less apparent socioeconomic gradient in incidences [11]. Furthermore, it is known that the inequalities observed with ecological measures are the same as those obtained with individual-based measures [46]–[47].

MEDEA is also a multidimensional measure of deprivation, making it likely to be more strongly associated with differences in health outcomes than are individual measures of poverty [15]. Moreover, there is a lack of standardization of deprivation scores and outcomes may vary depending on which deprivation index is used; nonetheless, the magnitude and direction of most of the associations between health outcomes and deprivation are maintained [1]. In our study, the relationships observed between the MEDEA index and cancer incidence appear to be robust.

In conclusion, deprivation was associated differently with the occurrence of various cancer types. These results provide evidence that MEDEA has good predictive validity [48], [49] and can be applied as an area-based deprivation index for use with the SIDIAP database, in accordance with previous studies [1], [11], [15]. Furthermore, the MEDEA index can identify areas in need of interventions and of additional primary care resources. Part of the association observed is explained by common, modifiable, lifestyle-related cancer risk factors and comorbidities. Therefore, public health policies should focus on effective ways to reach patients with proven cancer prevention and management strategies such as smoking cessation, responsible use of alcohol, and healthy dietary behaviors, particularly in economically deprived urban areas.
